# Systematic identification of an integrative network module during senescence from time-series gene expression

**DOI:** 10.1186/s12918-017-0417-1

**Published:** 2017-03-15

**Authors:** Chihyun Park, So Jeong Yun, Sung Jin Ryu, Soyoung Lee, Young-Sam Lee, Youngmi Yoon, Sang Chul Park

**Affiliations:** 1 Well-Aging Research Center, Samsung Advanced Institute of Technology, Samsung Electronics, Suwon, South Korea; 2 Biomedical HPC Technology Research Center, Korean Institute of Science and Technology Information, Daejeon, South Korea; 3 Department of New Biology, DGIST, Daegu, South Korea; 4 Department of Computer Engineering, Gachon University, Seongnam-si, South Korea; 5 Mobile Healthcare Lab., Samsung Advanced Institute of Technology, Samsung Electronics, Suwon, South Korea

**Keywords:** Senescence, Time-series gene expression, Data integration, Network analysis

## Abstract

**Background:**

Cellular senescence irreversibly arrests growth of human diploid cells. In addition, recent studies have indicated that senescence is a multi-step evolving process related to important complex biological processes. Most studies analyzed only the genes and their functions representing each senescence phase without considering gene-level interactions and continuously perturbed genes. It is necessary to reveal the genotypic mechanism inferred by affected genes and their interaction underlying the senescence process.

**Results:**

We suggested a novel computational approach to identify an integrative network which profiles an underlying genotypic signature from time-series gene expression data. The relatively perturbed genes were selected for each time point based on the proposed scoring measure denominated as perturbation scores. Then, the selected genes were integrated with protein-protein interactions to construct time point specific network. From these constructed networks, the conserved edges across time point were extracted for the common network and statistical test was performed to demonstrate that the network could explain the phenotypic alteration. As a result, it was confirmed that the difference of average perturbation scores of common networks at both two time points could explain the phenotypic alteration. We also performed functional enrichment on the common network and identified high association with phenotypic alteration. Remarkably, we observed that the identified cell cycle specific common network played an important role in replicative senescence as a key regulator.

**Conclusions:**

Heretofore, the network analysis from time series gene expression data has been focused on what topological structure was changed over time point. Conversely, we focused on the conserved structure but its context was changed in course of time and showed it was available to explain the phenotypic changes. We expect that the proposed method will help to elucidate the biological mechanism unrevealed by the existing approaches.

**Electronic supplementary material:**

The online version of this article (doi:10.1186/s12918-017-0417-1) contains supplementary material, which is available to authorized users.

## Background

Cellular senescence is irreversible exit from the cell cycle resulting from the limited replicative capacity [1] caused by telomere shortening [2, 3], DNA damage, and the epigenetic derepression of several genes such as the *IKK4a/ARF* locus [4]. Senescence and aging are complex processes with multiple causal mechanisms [5]. Recently, due to increase of understanding for the senescence mechanism, it was shown to be a heterogeneous phenotype driven by multiple casual mechanisms instead of a singular state [6]. To understand senescence, an analytical approach based on systems biology is needed to reveal the interactions among several multiple effector programs of the senescence phenotype [7]. Genome-wide profiling of molecular-level changes during senescence such as changes in gene expression is particularly important.

A recent study analyzed genome-wide gene expression at various time points during the establishment of replicative senescence and revealed senescence stage-specific gene perturbations [8]. Analysis of the functional enrichment for each stage indicated initial perturbation of cell cycle-related genes and subsequent perturbation of metabolic, inflammatory, and immune-related genes at the middle stage. At the final stage, genes related to cell death and cell growth regulation were perturbed. Thus, genome-wide time-series analysis can reveal the genotypic signature underlying senescence.

Time-series gene expression data have been widely used to explore the molecular-level events during a phase change such as the senescence process described above, despite of difficulty of culturing cells to full senescence. Typical analytical methods use co-expression patterns to identify functional modules or compare pairwise time points to capture features of the transition or to identify temporally regulated gene expression versus one control sample [9]. Consequently, these approaches yield results based on individual genes or gene sets without considering the connectivity between them. However, cellular processes involve the interactions among several molecules, and these processes can be represented as a biological network with genes or proteins as nodes and their relationships as edges. Thus, interactome data of biophysically interacting proteins are especially useful for analyzing biological processes, but few attempts have been made to integrate time-series gene expression and protein-protein interaction (PPI) data, particularly in senescence and aging.

Recently, a study reported the construction of an age-specific integrative gene network with PPI and topological analysis of the network to reveal the key modules in aging [10]. To construct a network, differentially expressed age-specific genes were selected by following methodology of [11], which had used criterion as follows; 1.5-fold change, 0.01 FDR. The protein interactions mapped with the selected genes become edges of the network. As a result, 37 age-specific networks were obtained and ‘ground truth’ gene set collected by analyzing brain gene expression data was used to demonstrate whether the networks were significantly related with the aging process or not. The authors revealed that the global topology of the age-specific networks was similar to each other, whereas the local topologies of several genes were significantly different. For the topological comparison among age-specific networks, the similarity measure called *graphlet degree distribution agreement* [12] was used. It was revealed that the local topologies were significantly changed with age and those genes were associated with age.

We proposed a novel approach to investigate the core modules of a genetic network highly correlated with phenotypic changes from time-series data. To construct the network, we integrated a perturbed gene set with biophysically validated PPIs recently published [13] and identified and analyzed the core sub-gene network. Based on the evidence that continuously perturbed interactions can be used to interpret a gradual phenotypic change with abrupt changes at the beginning and end points, we hypothesized that a sub-gene network with a constant topological structure but gradually altered context (e.g., expression pattern) plays an important role in phenotypic change. This concept can be applied to study cellular senescence because several senescence-related functions, such as the cell cycle, are not enriched at the abrupt phase; actually, a previous study revealed that cell cycle remodeling was nearly continuous during senescence [14]. The proposed approach is distinguished from the previous senescence studies. The previously mentioned study [8] did not consider the interactions among the perturbed genes during senescence and [10] analyzed the network modules topologically changed during aging.

To test our hypothesis, we applied the proposed method to two replicative senescence datasets from human diploid fibroblasts (HDFs) and mesenchymal stem cells (MSCs) and one independent cancer progression dataset from human tissue neoplasia. We performed functional enrichment of the identified core sub-gene network and simple significance tests to confirm whether our findings reflect changes in gene expression that account for the observed phenotypic change.

## Methods

### Data description and system overview

As a proof of concept for our approach, we intensively used recently published time-series gene expression data (GSE41714) measured during replicative senescence in HDFs [8]. This time-series microarray data (GSE41714) was composed of twelve sequential order of senescence stages according to the population doubling time including young and old phenotypes as the first and last time points, respectively. The senescence phenotype for each stage was identified and confirmed by typical determinants such as increased or decreased reactive oxygen species (ROS) levels and high or low level of senescence-associated β-galactosidase activity. We also used time-series microarray data (GSE9593) collected during replicative senescence in MSCs [15]. These data included nine passages from young to old status. Finally, we employed time-series microarray data (GSE15299) for elucidating epithelial cancer progression [16]. This dataset included four time points and was selected to test whether the proposed approach was applicable to processes besides senescence. The series matrix files of these microarray based data were downloaded and if there was no obvious description about normalization process in GEO, the normalization was performed. The array platform of these three datasets was different each other. GSE41714, GSE9593 and GSE15299 used Illumina HumanHT-12 V4.0 expression beadchip, Illumina HumanHT-12 V4.0 expression beadchip and Affymetrix Human Genome U133 Plus 2.0 Array, respectively.

To identify the connectivity between genes, we downloaded a recently published human PPI dataset [13] with 23,124 high-confidence PPIs compiled from systematic screening with high-throughput yeast two-hybrid and literature studies and validated using biological assays. Although the high-confidence PPI set is smaller than the typical PPI set from the I2D database [17–19], biological validation allows greater confidence in conclusions based on these PPIs. The proteins in these PPIs were mapped into gene symbols using UniPROT [20].

**Fig. 1:**
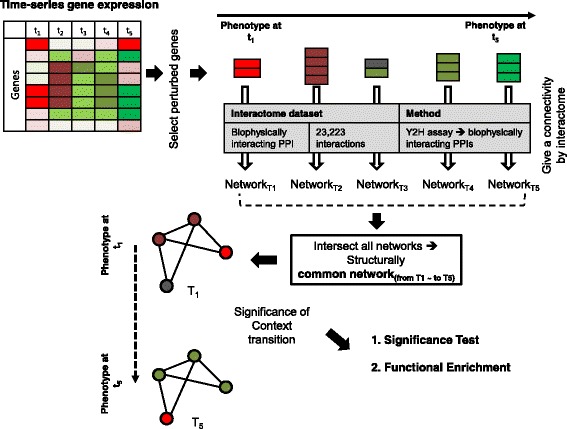
Overall workflow of the proposed method. For each time point, interactions including perturbed genes are identified from protein-protein interactions, and networks are constructed. The common network across time points is identified and it is validated by performing computational and functional significance tests

The entire workflow of the proposed method is shown in Fig. 1. As described above, time-series gene expression dataset and PPIs are integrated and the structurally conserved network is identified. Using this network, phenotypic changes during senescence and cancer progression are analyzed.

### Identification of time-specific networks

Before identifying the perturbed gene set, we carry out quantile normalization for the time-series gene expression dataset with R. For normalization of the downloaded dataset which is series matrix format in GEO, we used ‘preprocessCore’ library provided by Bioconductor. By normalization, we can expect that the average of expression values and relatively highly expressed genes at certain time points were adjusted. This gives a similar intensity distribution for each time point. After normalization, perturbed gene sets were identified to construct a network for each time point. In our method, the perturbation score of a gene at a time point is calculated as follows:

$$ p\left( gen{e}_{i j}\right)={e}_{i j}-\frac{1}{M}\sum_{k=1}^M{e}_{i, k} $$

where *gene*_ij_ indicates gene *i* at time point *j*, *e*_ij_ indicates the expression value of the *i* ^th^ gene at the *j* ^th^ time point, and M is the total number of time points. This formula measures the difference between each expression value and the average for expression value. The perturbation score, *p*(*gene*_ij_), is calculated for every gene at every time point, and larger values implied that gene *i* was relatively perturbed at time point *j*. If adjacent time points with similar phenotypes are grouped, the average perturbation score for the grouped time points was used.

**Fig. 2:**
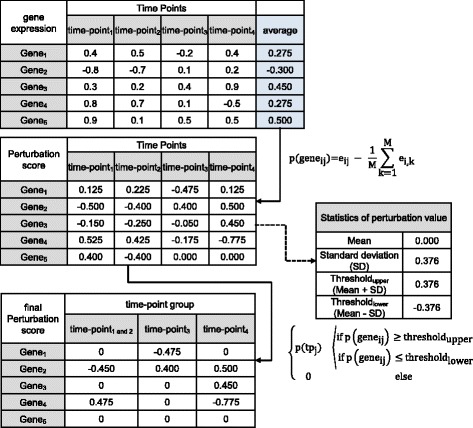
Identification of temporally perturbed genes. In the time-series microarray, each expression value is converted into a perturbation score. After establishing a threshold value from these perturbed scores, filtered perturbed scores satisfying thresholding condition are employed to select interactions

Only genes with perturbation scores above the threshold (minimum cut-off value for significance of perturbation) are selected for each grouped time point. The threshold as determined for a given dataset by assuming the perturbation scores are normally distributed and setting the threshold as the sum of the mean and standard deviation. Figure 2 depicts a detailed toy example of the entire analytical flow to identify perturbed genes. First, the average expression values are calculated for five genes. Then perturbation score for each gene at each time point is calculated. For example, the perturbation score of the third gene at time-point 2 is (0.2–0.45) = −0.25. Final perturbation scores of the third gene at merged time-point 1, 2 are calculated as follows; if time point 1 and 2 can be grouped, the average perturbation score is−0.2 calculated by (−0.15–0.25)/2. Then this score is compared with upper and lower threshold values (0.376 and−0.376, respectively). In this example, the final perturbation score is 0 because the average perturbation score is more than lower threshold and less than upper threshold. If the final perturbation score of the gene is not zero, this gene can be included in the perturbed gene set for each time point. To construct a time point-specific network, we identify a set of interactions including at least one perturbed gene from the high-confidence PPI dataset. From these selected interactions for each time-point, time-specific networks are built.

### Identification and analysis of common network

From the identified time-specific networks which have different size and include different interactions, we detect topologically conserved sub-network across time-point. As described in the Introduction, we assumed that sub-networks with constant topological structures play important roles in phenotype change. Topological conservation for a sub-network refers to its continuous perturbation with changing phenotype. As mentioned in the Introduction, we assumed that these network modules and the transition of their information such as perturbation scores were related to phenotypic changes. To prove our assumption, we calculate the average difference of perturbation scores between two time points after identification of common network. For example, in Fig. 2, if gene 2 and gene 4 are selected as a member of common network, the average difference of perturbation scores between time-point_1and2_ and time-point_4_ will be 1.1 which is calculated by (|0.5–(−0.45)| + |-0.775–0.475|)/2. From this calculation, we can determine whether the variation of perturbation scores and the state of phenotypic changes are associated or not. To investigate that perturbation scores were related to phenotypic changes, the statistical tests for all possible pairs of adjacent time points and one additional pair composed of the first and the last time points was performed.

### Statistical support for significance of common network

**Fig. 3:**
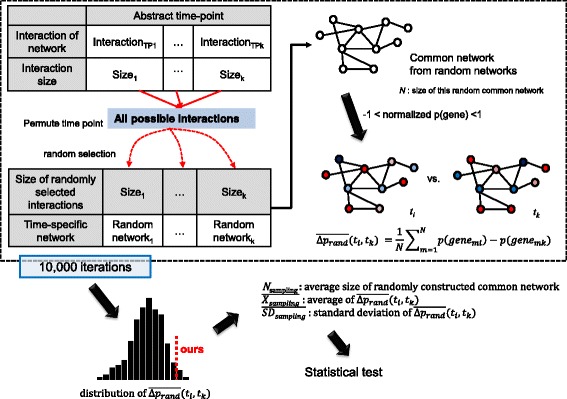
Workflow of computational validation. As many as the number of interactions for each time point identified by the proposed method, interactions are randomly selected without considering their time points to construct random networks. From these random networks, a common network is identified. At two time points which can explain phenotypic change, the average difference of perturbation scores is calculated on common network. This random sampling process is repeated as many as 10,000 iterations. Based on the distribution from 10,000 average differences of perturbation scores, the statistical significance of our average difference of perturbation score is performed using statistical test

We investigate an association between phenotypic changes and variation of perturbation scores with statistical test based on large-sized random sampling. We assume ‘average differences of perturbation scores from our common network and random network are same’ as a null hypothesis. We tested whether this hypothesis can be rejected or not. The test is composed of three steps. As shown in Fig. 3, first, all interactions identified by the proposed method are collected. From this collection, the interactions are randomly chosen as numerous as the proposed method selected. For example, if 5 interactions were identified by the proposed method at time point 2, the same-sized 5 interactions are randomly selected from the collection about this time point. Consequentially, the random selection only permutes order of time point. Secondly, across time point, common network is detected from randomly selected interaction set. Lastly, we calculate the average difference of perturbation scores between two time points with this common network. These three steps are repeated 10,000 times to generate the distribution. We calculate *p*-value which indicates whether we can reject the hypothesis or not. Through the result of this test, we demonstrate that context transition explainable phenotypic change can be observed in the conserved network reflecting a time order, i.e. identified by the proposed method. This procedure was systematically implemented by using Java and R.

## Results

To demonstrate the effectiveness of the proposed method, we applied it to three time-series gene expression datasets: two senescence datasets and a cancer progression dataset. Although we focused on senescence, we included the cancer progression dataset to test the applicability of our method to other types of datasets. We detected a common network from whole time point-specific networks and demonstrated how the common network can explain the phenotypic change for the three experimental datasets by performing statistical tests and functional enrichment with KEGG pathway database and gene ontology database.

To determine a optimal thresholds for identifying perturbed genes, we used dataset-specific cut-off values. As described in Method section, we calculated the mean and standard deviation for the perturbation scores in given dataset and found that the perturbation scores were not normally distributed: the mean was near zero, and the standard deviation was relatively small. However, we assumed that the distribution was Gaussian because the number of perturbation scores was large enough to apply the central limit theorem. Supporting Fig. 6 shows the distribution about perturbation scores. We used μ ± 1σ as a threshold: perturbation scores above μ + 1σ or below μ–1σ were chosen to construct each network. The number of genes selected by using μ ± 2σ or μ ± 3σ as a threshold was too small to construct networks. The experimental results while changing the cut-off threshold were shown in supporting Table 4 and 5 of the additional file.

### Significance test in HDF senescence and cancer progression dataset

**Fig. 4:**
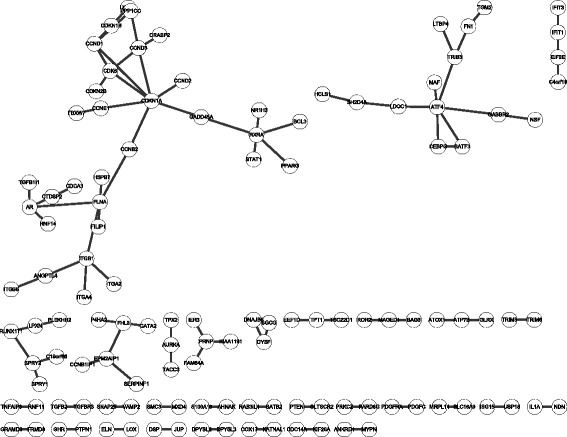
Visualization of common network of HDF senescence dataset

Table 1 summarizes the identified time-specific networks and their common network in the replicative senescence dataset (GSE41714) [8]. Twelve time points based on the population doubling time were grouped into four stages according to the senescence phenotype. The network size was different at each stage, and the proportion of selected interactions was less than 11.2% in general; the common network comprised only ~0.409% of all used high-confidence PPIs. We assumed that this network might act as a key module for inducing phenotypic changes despite its small size. Figure 4 shows the identifed common network.

**Table 1:** Network information about the stage-specific network

Network	No. of node	No. of edge	Ratio of the used interaction (%)
Early stage	1,748	2,602	11.200
Middle stage	650	701	3.017
Advanced stage	302	266	1.150
Very advanced stage	1,085	1,448	6.233
Common network	122	95	0.409

Threshold: ±0.2046

Total number of used interactions for constructing network: 23,124

With the common network, we investigated a statistical significance comparing all possible adjacent stages. As shown in Table 2, among three possible adjacent comparisons, only the second comparison (middle-advanced) was significant. From this result, we could suggest that phenotypic change between middle and advanced stages was relatively more influential than other transitions. Actually, it was reported in the previous study [8] that the results of functional analysis in early and middle stage were similar each other. These two stages were highly related with cell cycle and metabolic process. Similary, the results of functional analysis in advanced and very advanced stage were similar. Previous study [8] reported these two stages were related with cell death and NFKB cascade functions. In addition, we performed the significant test for comparison between early and very advanced stage, which indicate the first and last, respectively. The *p*-value was almost zero for this comparison. The phenotypic changes between the first and last stage was understandably obvious since young cell turned into old cell. This result demonstrated that context transition of the conserved network can reflect and explain the phenotypic changes. Surpporting Fig. 3 shows the four distributions of average difference of perturbation scores from random samplings for each comparison. In additon, we carried out multiple comparison tests with final perturbation scores of the selected genes for each time-point using ANOVA and TukeyHSD method in R. In this experiments, we identified that the early and very advanced time point was most significantly different. Supporting Table 6 shows this results.

**Table 2:** The result of statistical significance test to compare perturbation scores between two time points on senescence dataset

Comparing time points	△P from our approach (mean)	△P from random sampling		*P*-value
		(mean)	(standard deviation)	
Early – Middle	0.326	0.309	0.062	3.679E-01
Middle – Advanced	0.655	0.205	0.075	3.159E-93
Advanced – Very advanced	0.180	0.165	0.048	3.081E-01
Early – Very advanced	0.940	0.501	0.092	2.725E-60

We performed statistical test with a significance level of 0.05. In two (early-middle, advanced-very advanced) among three adjacent comparisons, the difference of perturbation score was not significant. However the difference of perturbation score at the comparison between early and very advanced time points was strongly significant

**Fig. 5:**
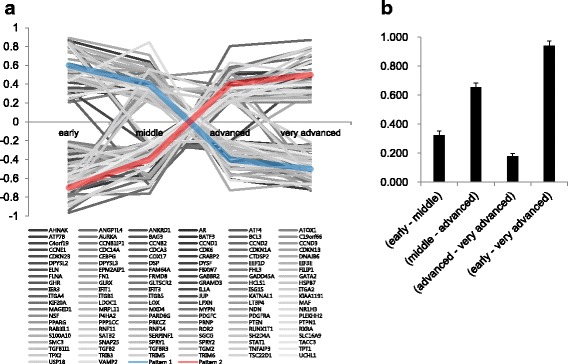
Profile on change of perturbation scores of the member genes in the identified common network (HDF senescence dataset). **a** Change of perturbation scores during senescence process. There were two striking patterns, one of which is gradually decreased pattern and the other is gradually increased pattern. **b** Comparison for changes of perturbation scores in adjacent two time points and the first and the last time point. We could identify that the changing pattern was reversed at both the first and the last time point. As a statistical result shown in Table 2, this reversely changed pattern of expression is rarely happen. Therefore, we could assume that the common network included information to explain the senescence process

We investigated how perturbation scores of the common network are changed during transition and whether this changes have direction to the increase or decrease. As shown in Fig. 5, we could find two representative patterns from changing profile of perturbation scores. Up and down fluctuation of these two patterns were almost opposed, mutually. We observed that relatively up-regulated genes in early stage were gradually decreased along with going to very advanced stage. On the contrary, relatively down-regulated genes in early stage were gradually increased along with senescence.

**Fig. 6:**
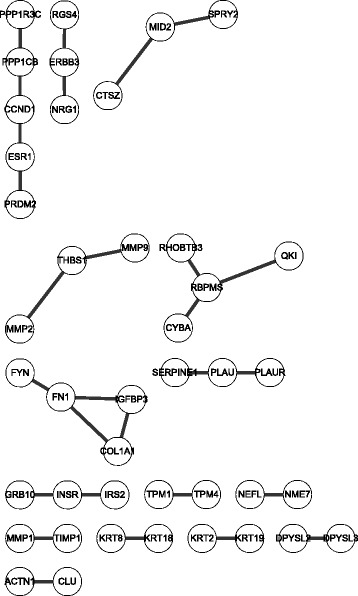
Visualization of common network of cancer progression dataset

Furthermore, we applied the proposed method to the cancer progression dataset (GSE15299) [16] and performed same experiments as mentioned above. Table 3 shows a summary of the identified networks for each stage. The network size was different at each stage, and the proportion of selected interactions was less than 10.2% in general; the common network comprised only ~0.12% of all used interactions. Figure 6 shows the identified common network. We investigated a statistical significance from all possible neighboring stages including a comparison between the first and last stage. As shown in Table 4, statistical test was also performed, we revealed that only one comparison between Day20 and Day 35 was not signficant.

**Table 3:** Network information for cancer progression dataset

Network	No. of node	No. of edge	Ratio of the used interaction (%)
Day 0	1462	2355	10.184
Day 5	390	383	1.656
Day 20	393	427	1.847
Day 35	534	626	2.707
Common network	42	28	0.121

Threshold: ±0.4205

Total number of used interactions for constructing network: 23,124

**Table 4:** The result of statistical significance test to compare perturbation scores between two time points on cancer progression dataset

Comparing time points	△P from our approach (mean)	△P from random sampling		*P*-value
		(mean)	(standard deviation)	
Day 0–Day 5	0.738	0.637	0.126	2.041E–02
Day 5–Day 20	0.715	0.289	0.119	3.240E–25
Day 20–Day 35	0.277	0.279	0.104	9.692E–01
Day 0–Day 35	1.315	0.743	0.148	3.792E–29

We performed statistical test with a significance level of 0.05. In one (Day 20 – Day 35) among three adjacent comparisons, the difference of perturbation score was not significant. However the difference of perturbation score at the comparison between Day 0 and Day 35 time points was strongly significant

**Fig. 7:**
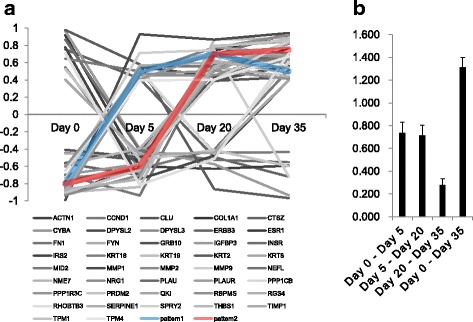
Profile on change of perturbation scores of the member genes in the identified common network (cancer progression). **a** Change of perturbation scores during cancer progression process. There were two striking patterns, they are gradually increased. **b** Comparison for changes of perturbation scores in adjacent two time points and the first and the last time point

To capture the progression of invasive neoplasia, the author made an Ras-inducible human model which can be changed epidermal tissue to squamous cell carcinoma [16]. In this experiments, hyperplasia and disordered tissue polarity were observed that between day 5 and day 10, and fully manifest invasive phenotype was culminated by day 25 to 30. Therefore, until day 20, phenotype was rapidly changed and after day 25, the fully changed phenotype was observed. Owing to this phenotypic change, the average difference of perturbation scores between day 20 and 35 was not significant compared to random case. We demonstrated that the common network constructed by our approach reflects this phenotypic changes. The *P*-value of comparision between the first and last day was 3.792E–29, which is considerably significant. As shown in Fig. 7, we also observed that the profile of perturbation scores in the common network could support the result of significant test.

### Functional enrichment in HDF senescence and cancer progression dataset

Through abovc experiments, we computationally analyzed common network in time-dependent gene expression profile. In addition to the computational validation, we performed two types of functional enrichment test on the common network. Fisrt, we performed gene ontology based enrichment using BiNGO [21] plugin in Cytoscape [22]. Secondly, we carried out pathway enrichment test using KEGG database. Because the identified common network had small size and simple topology, we used the entire common network as an input of functional enrichment test.

On HDF senescence dataset, top 10 pathways and gene ontology terms (*P*-value < 0.01) were listed in Table 5. As expected, among several pathways, cell cycle and cancer related pathway were significantly enriched. In gene ontolgies, response to several stimulus, cell proliferation and development related processes were importantly enriched. These processes were regarded as an important roles in senescence. On cancer progression dataset, we also selected top ten enriched pathways and gene ontology terms. The result was described in Table 6. In KEGG pathways, bladder cancer pathway was enriched. In gene ontologies, cancer progression associated term was enriched such as anatomical structure development.

**Table 5:** On the common network from HDF senescence dataset, the list of top 10 terms of functional enrichment test with KEGG pathway and Gene Ontology database. (*P*-value < 0.01)

Category	Term	-Log_10_ (*P*-value)
KEGG pathway	Cell cycle	8.292
	Pathways in cancer	7.553
	Small cell lung cancer	6.699
	p53 signaling pathway	6.301
	Focal adhesion	6.000
	Prostate cancer	5.398
	Arrhythmogenic right ventricular cardiomyopathy (ARVC)	3.824
	Melanoma	3.032
	Hypertrophic cardiomyopathy (HCM)	2.678
	Dilated cardiomyopathy	2.523
Gene Ontology (Biological Process)	response to endogenous stimulus	11.780
	regulation of cell proliferation	11.513
	negative regulation of cell proliferation	11.209
	response to hormone stimulus	10.919
	organ development	10.213
	response to organic cyclic substance	10.052
	negative regulation of epithelial cell proliferation	9.901
	response to steroid hormone stimulus	9.870
	response to organic substance	9.744
	system development	9.547

**Table 6:** On the common network from cancer progression dataset, the list of top 10 terms of functional enrichment test with KEGG pathway and Gene Ontology database. (*P*-value < 0.01)

Category	Term	-Log_10_ (*P*-value)
KEGG pathway	Bladder cancer	2.469
Gene Ontology (Biological Process)	organ development	11.150
	system development	10.105
	anatomical structure development	10.069
	anatomical structure morphogenesis	9.562
	regulation of glucan biosynthetic process	9.138
	regulation of polysaccharide biosynthetic process	9.138
	regulation of glycogen biosynthetic process	9.138
	response to chemical stimulus	9.099
	response to endogenous stimulus	9.073
	regulation of polysaccharide metabolic process	8.988

### Additional analysis with MSC senescence dataset

We performed statistical test and functional enrichment for MSC senescence dataset. The detailed results were described in the Additional file 1 with supporting Table 1, 2 and 3 and supporting Figs. 1 and 2. The distribution of average difference between all pairs of two time point used in our test was shown in Additional file 1 (supporting Figs. 3, 4 and 5). The information of the identified common networks from three datasets was listed in Additional file 2.

### Identification of regulatory module from common network

Time point-specific gene networks can be more important than the common network in most analyses. However, our approach aids in elucidating temporal changes in biological functions. Thus, our approach is appropriate for investigation of continuously affected molecular effectors such a cell cycle in replicative senescence. To demonstrate this hypothesis, we performed additional experiments and described the result herein.

**Fig. 8:**
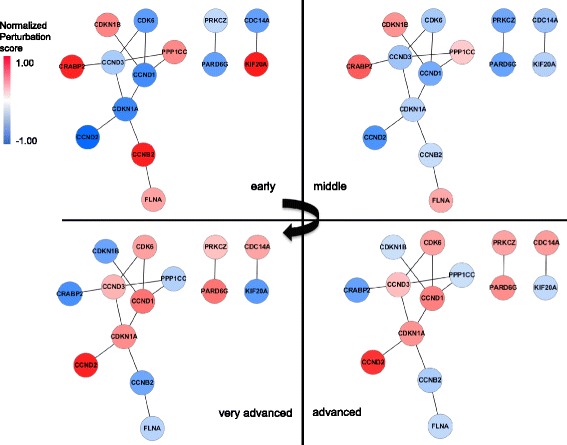
Visualized dynamic change of the perturbation scores in the identified common network limited to cell cycle-related genes

We attempted to trace temporal changes in the pattern of perturbation scores for the cell cycle specific common network. To identify this common network from HDF senescence dataset, we only used and focused on 207 known cell cycle-related genes which are annotated in the gene ontology database. We identified a common network comprising 14 nodes and 13 edges as shown in Fig. 8. Among the 207 cell cycle-related genes, only 14 continuously affected senescence while maintaining a constant topology and displaying gradual and directional changes in perturbation score values. During aging, we identified genes known to be up- and down-regulated [23–25] during senescence with similar regulations in our analysis. For example, KIF20, a cell cycle controller, was down-regulated in senescence status by activation of p16 via the Rb/E2F pathway [23]; CRABP2 was strongly down-regulated with increased passage number in human amniotic fluid-derived stem cells and might act as a negative regulator to limit cellular senescence [24]; and CCND1, another well-known cell cycle regulator, is down-regulated in HDF senescence [25].

As has been demonstrated by several previous studies [23–25], variation of the perturbation score values in the identified common network was generally consistent with expectations for cellular senescence. Interestingly, the gene expressions at the young and senescence stage were completely opposed. Based on this result, we propose that the identified common network can switch cell cycle activity between young and senescence status. Alternatively, transcription factors (TFs) that regulate genes in the identified common network could act as the switch.

**Fig. 9:**
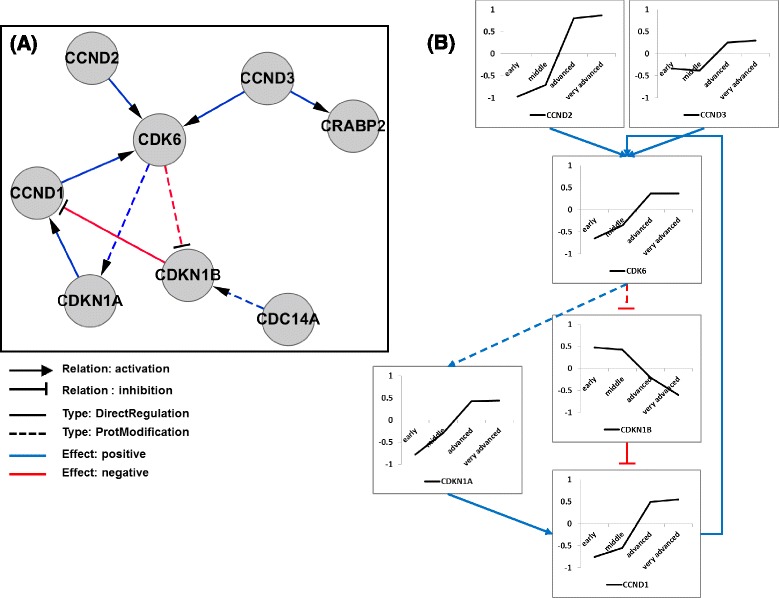
Inferred regulatory relationship and perturbation score changes according to the senescence process. **a** Regulatory pathway of the identified cell cycle-specific common network constructed using PathwayStudio. **b** Transition of the expression level reflecting the regulatory relationship among member genes in (A)

In this study, we focused on the former assumption because there may be regulatory relationships among genes of the network. We attempted to identify the regulatory relationships for the identified common network using PathwayStudio 9.0 software (Ariadne Genomics; Rockville, MD, USA) to build a pathway of directed interactions among genes in common network (Fig. 9a), omitting six orphan genes. We included two types of regulation: ProtModification and DirectRegulation. ProtModification indicates a regulator that modifies the target molecule through phosphorylation, glycosylation, etc., and DirectRegulation indicates direct physical interactions that can influence target activity.

Interestingly, we observed a feedback loop composed of the replicative senescence related genes: CDK6, CCND1, CDKN1A, and CDKN1B (Fig. 9a). In the observed regulatory network, including the feedback loop, CDK6 was the most important node because it acted as a hub and could be the genesis of the loop. It has been known that CDK6 regulates DNA replication in G1 and reported to switch cell cycle status from G1 phase to S phase [26]. Furthermore, high expression of CDK6 can form a transcription complex and induce the expression of tumor suppressor p16 [27]. It has been reported that p16 is significantly related with molecular mechanism of senescence [28].

In our feedback loop (Fig. 9b), the subunit regulators of CDK6 (CCND1, CCND2, and CCND3) were down-regulated in young status; thus, these four proteins were simultaneously down-regulated in young status. However, expression of CDK6 was increased upon senescence. Along with this expression alteration of CDK6, CDK6 could inhibit CDKN1B by negatively regulating its phosphorylation. CDKN1B translation is also reduced during G1 arrest [29], and CDKN1B down-regulation could inhibit CCND1, which is also positively influenced by CDKN1A. Thus, CDK6 may be consistently up-regulated by CCND1 during senescence, allowing maintenance of full senescence status. Much of this process has been previously reported [30–32], but our method allowed identification of the regulatory relationship among them with a more integrated view, and we note that our approach yielded a result reflecting the senescence process.

In addition, we investigated TFs which can control this regulatory relationship. We used recently published computational method, iRegulon [33]. Among the results which have high enrichment score (NES > 0.3), we selected TFs inferred by TRANSFAC database widely used to search TFs. Then, we filtered TFs targeting on CDK6. As a result, we can identified seven TFs; STAT5A, ARID3A, POU4F3, DLX5, ZNF35, LMX1A, PAX2. Among them, STAT5A and ARID3A have been revealed to be related with cell cycle process [34, 35]. PAX2 has been reported to be mechanistically associated cancer cell proliferation [36]. Through this result, it is possible that the identified regulatory relationship and the related TFs can be regarded as strong candidate which controls cell cycle phase replicative senescence.

## Discussion

**Fig. 10:**
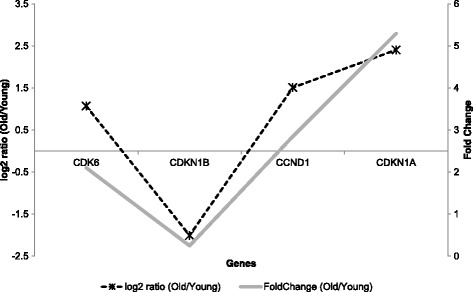
Relative gene expression profile and fold change values about four genes that compose the identified feedback loop. CDK6, CCND1 and CDKN1A were up-regulated in old status. On the other hand, CDKN1B was down-regulated in old status. This expression pattern was corresponding to the transition pattern for gene expression of the regulatory feedback loop during senescence

We analyzed whether the expression levels of four genes constructing above feedback loop are corresponding to the regulatory loop or not using an independent dataset. We used recently published gene expression profile on the replicative senescence in normal human diploid fibroblasts [37]. In this dataset, young and old status was defined as being less than 40 population doublings and more than 70 population doublings, respectively. The relative gene expression level was measured by comparing young and old status. Through analysis of this profile, as shown in Fig. 10, we observed that expression pattern of the independent dataset follows the changing relation of the identified regulatory feedback loop according to senescence. Based on the analysis, we carefully expected that the identified regulatory module can be a part of cell cycle modulation, if we limited to HDF cell. We are planning to apply our method to tissue level gene expression profile in order to identify a module controlling aging process.

## Conclusions

In this study, we proposed a novel approach to identify gene networks that are significantly correlated with phenotypic changes from time-series data. In this process, we integrated a recently published PPI dataset with time-series gene expression data to produce informative interactions among genes. Networks were validated with statistical tests and functional enrichment. To demonstrate the suitability of the proposed method, we used three different real datasets for cellular senescence and cancer progression. The identified networks were appropriate to explain the phenotypic changes. In our future work, we plan to carry out perturbing experiments with the identified TFs to demonstrate whether they can contribute to changing phenotype by affecting expression level of CDK6 and its looping member or not.

## Additional files

Additional file 1: The experimental results with MSC senescence dataset (DOC 708 kb)
Additional file 2: List of the identified common network information (XLSX 30 kb)

## Abbreviations

FDR: False discovery rate
HDF: Human diploid fibroblasts
MSC: Mesenchymal stem cells
PPI: Protein-protein interaction
ROS: Reactive oxygen species
TFs: Transcription factors
