# Supplementary material for: Systematic identification of an integrative network module during senescence from time-series gene expression
Source: BMC Syst Biol. 2017 Mar 15;11:36. doi: 10.1186/s12918-017-0417-1 (PMC5353876; doi:10.1186/s12918-017-0417-1)
Supplement: Additional file 1: — The experimental results with MSC senescence dataset (DOC 708 kb) [file 12918_2017_417_MOESM1_ESM.doc]

**Systematic identification of an integrative network module during senescence from time-series gene expression**

Chihyun Park^1,2^, So Jeong Yun^1^, Sung Jin Ryu^1^, Soyoung Lee^1,5^, Young-Sam Lee^1,3^, Youngmi Yoon^4†^ and Sang Chul Park^1,3†^

1. Well-Aging Research Center, Samsung Advanced Institute of Technology, Samsung Electronics, Korea

2. Biomedical HPC Technology Research Center, Korean Institute of Science and Technology Information, Korea

3. Department of New Biology, DGIST, Korea

4. Department of Computer Engineering, Gachon University, Korea

5. Mobile Healthcare Lab., Samsung Advanced Institute of Technology, Samsung Electronics, Korea

Chihyun Park, Ph.D. Email: [tianelltio@gmail.com](mailto:tianelltio@gmail.com)

So Jeong Yun, Ph.D. Email: [sojeongyun@gmail.com](mailto:sojeongyun@gmail.com)

Sung-Jin Ryu, Ph.D. Email: [sungjin.ryu@samsung.com](mailto:sungjin.ryu@samsung.com)

Soyoung Lee, Ph.D. Email: [somuz.lee@samsung.com](mailto:somuz.lee@samsung.com)

Young-Sam Lee, Ph.D. Email: [lee.youngsam@dgist.ac.kr](mailto:lee.youngsam@dgist.ac.kr)

Youngmi Yoon, Ph.D. Email: [ymyoon@gachon.ac.kr](mailto:ymyoon@gachon.ac.kr)

Sang Chul Park, MD, Ph.D. Email: [blueocean2016@dgist.ac.kr](mailto:blueocean2016@dgist.ac.kr)

^†^Co-correspondence author

**Results of significance test and functional enrichment in MSC senescence dataset**

We applied the proposed method to the MSC dataset (GSE9593) [1]. Supporting Table 1 summarizes the identified networks. Nine passages were merged into four groups based on hierarchical clustering from the original study [1]. The network size was different at each stage, and the proportion of selected interactions was generally less than 10.5%. We identified a common network composed of 37 nodes and 23 edges (~0.1% of all interactions), smaller than the replicative senescecne dataset. We investigated a statistical significance with all possible neighboring comparisons from four time point including a comparison between the first and last stage. As shown in supporting table 2, statistical test was also performed, we revealed that all comparisons was signficant. In this dataset, almost perturbation scores of the common nework were flucuated up and down along with senescence process. As shown in supporing figure 2, two patterns were remarkably represented and they were opposed each other and almost perturbation scores from early to late time point were increased or decreased. Actually, going from passage 2,3 to passage 10, 11, the authors observed phenotypic changes and analyzed its impact. In addition, by performing KEGG pathway enrichment and gene ontology enrichment, we observed that the common network was significantly related with cell cycle process. The detailed results were listed in supporting table 3.

**Figures and Tables**


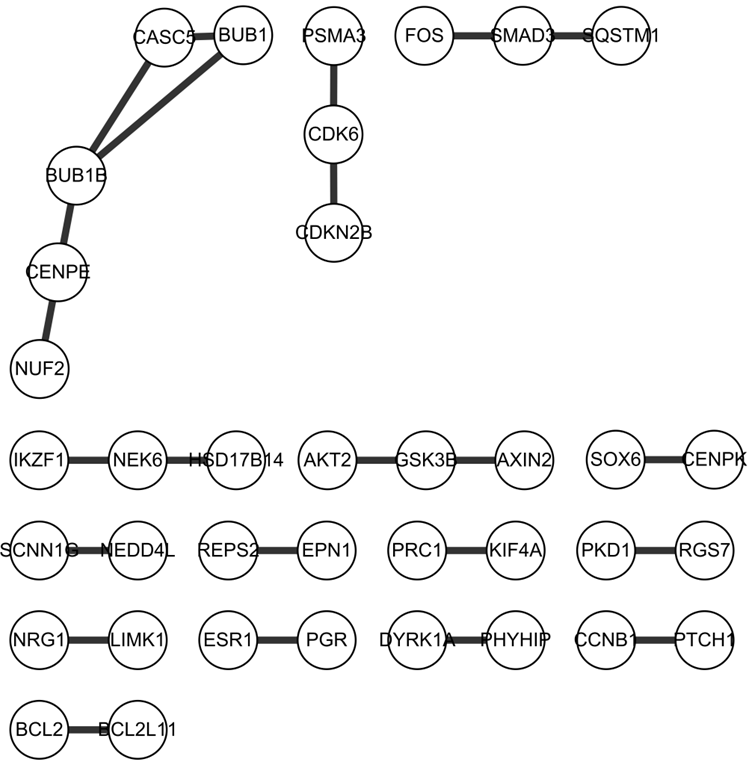


Supporting Figure 1. Visualization of common network of MSC senescence dataset


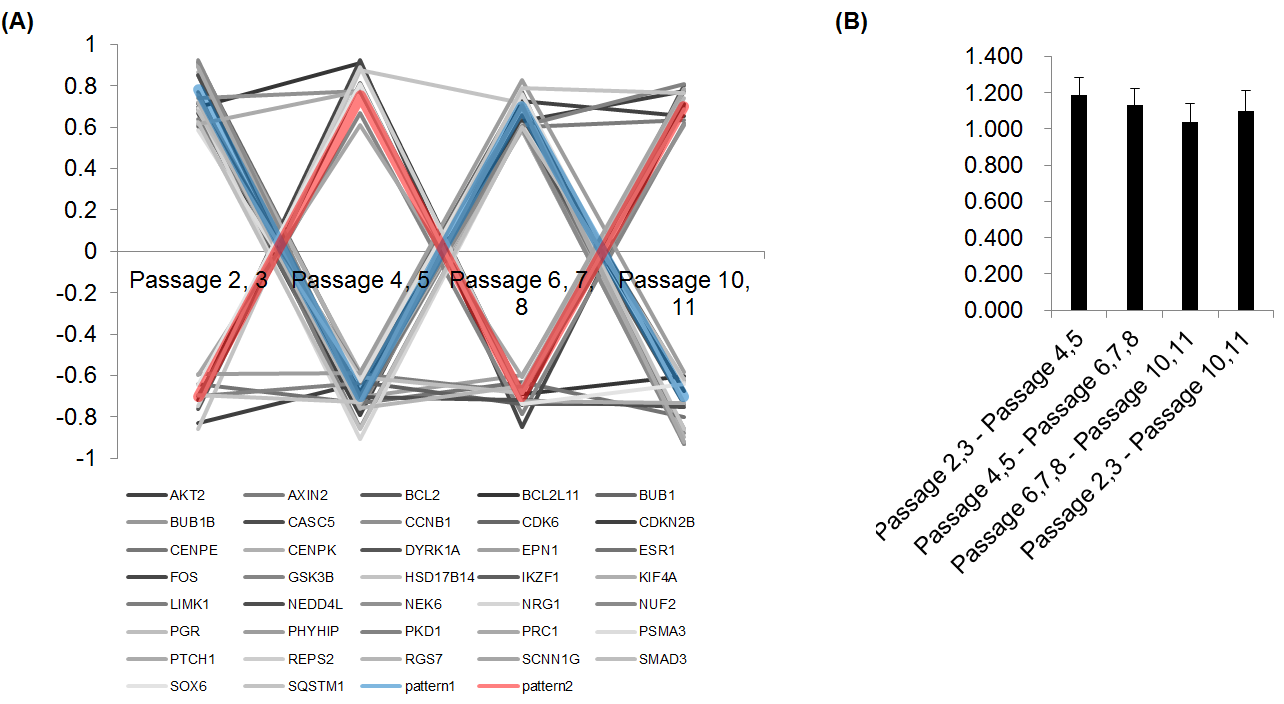


Supporting Figure 2. Profile on change of perturbation scores of the member genes in the identified common network (MSC senescence dataset). (A) Change of perturbation scores during senescence process. There were two striking patterns where the perturbation scores fluctuated between high and low. This two patterns were almost reversed for each other. (B) comparison for changes of perturbation scores in adjacent two time points and the first and the last time point. We could identify that the changes in all comparisons was similar and high. In spite of the fluctuation of perturbation scores for each time point, the changes between the first and the last time point were distinctly increased or decreased.


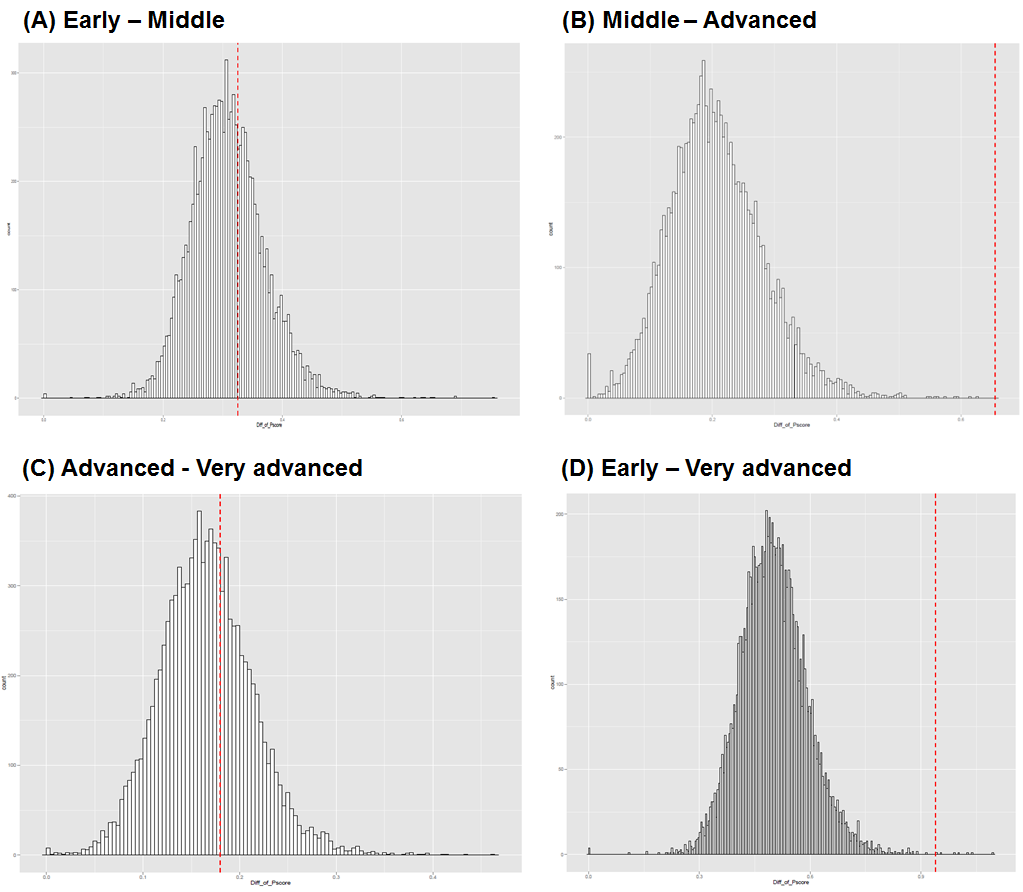


Supporting Figure 3. In each time-point comparison of HDF senescence dataset, the distribution of the average difference of perturbation scores from random constructed common network. The red dashed line indicates the average difference of perturbation scores calculated by


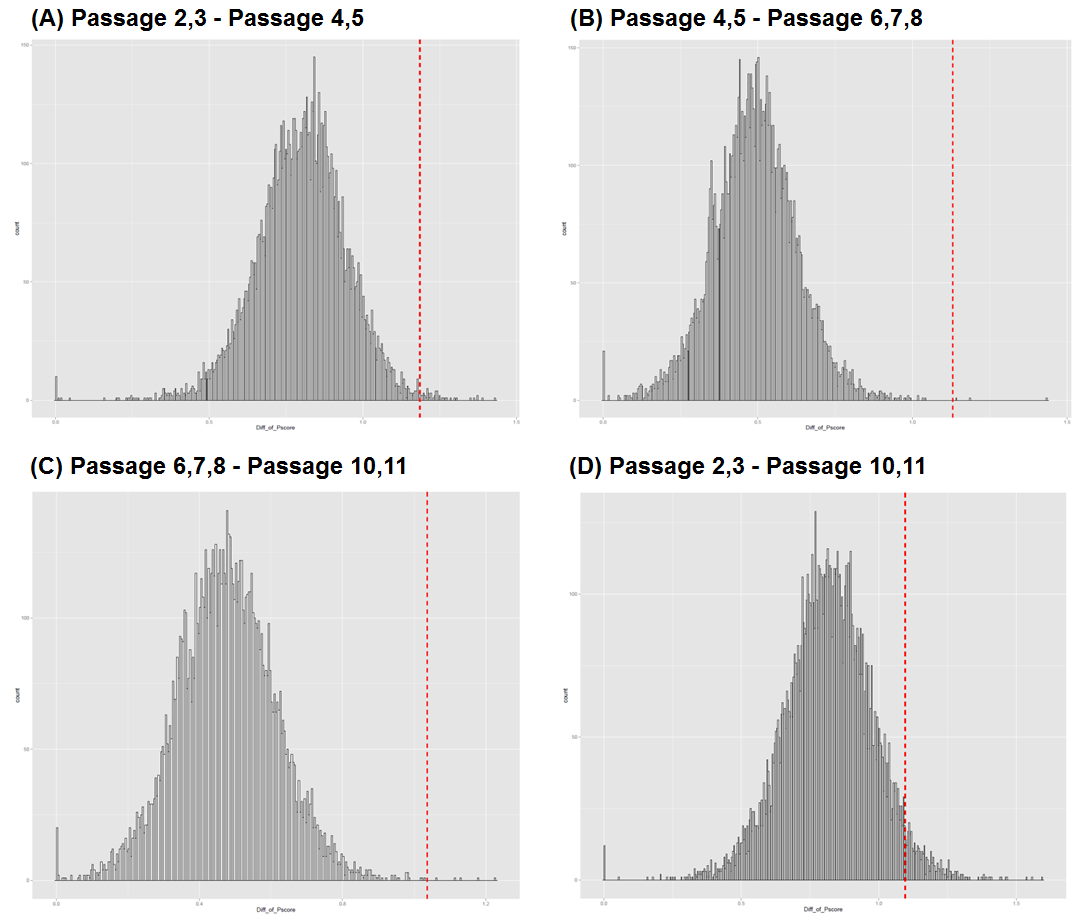


Supporting Figure 4. In each time-point comparison of MSC senescence dataset, the distribution of the average difference of perturbation scores from random constructed common network. The red dashed line indicates the average difference of perturbation scores calculated by our approach.


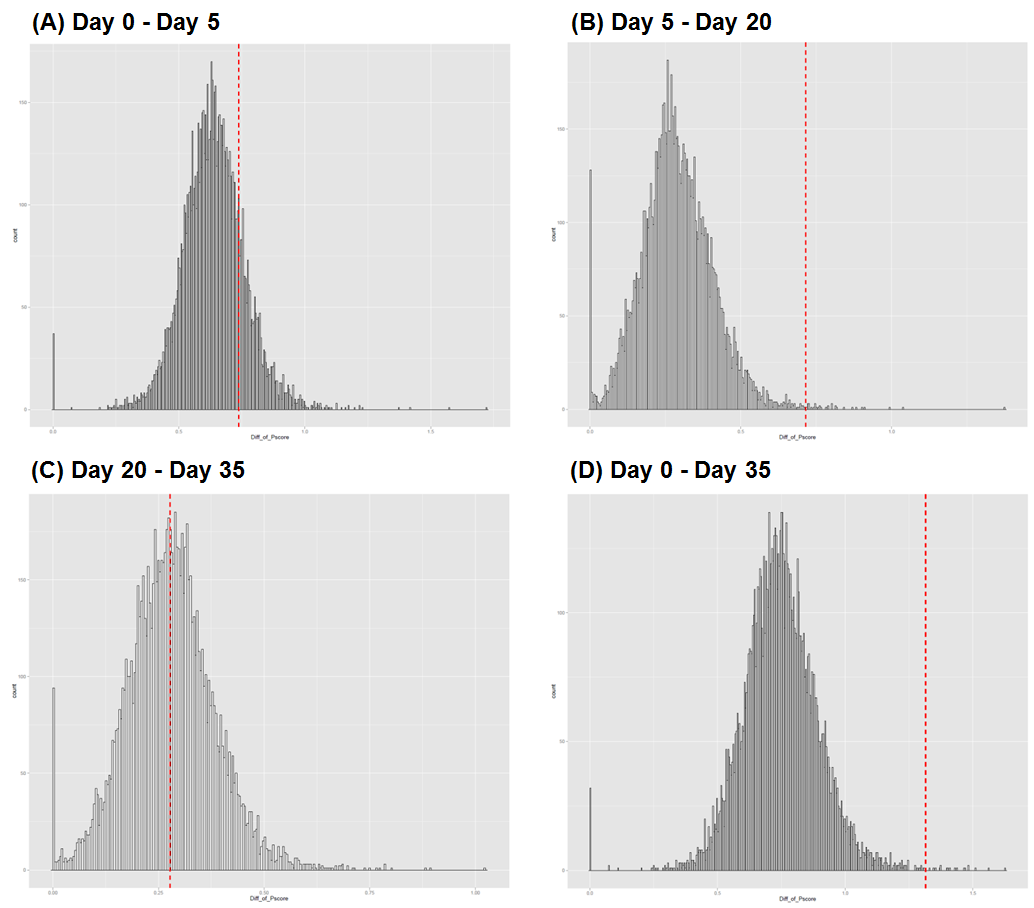


Supporting Figure 5. In each time-point comparison of cancer progression dataset, the distribution of the average difference of perturbation scores from random constructed common network. The red dashed line indicates the average difference of perturbation scores calculated by our approach.


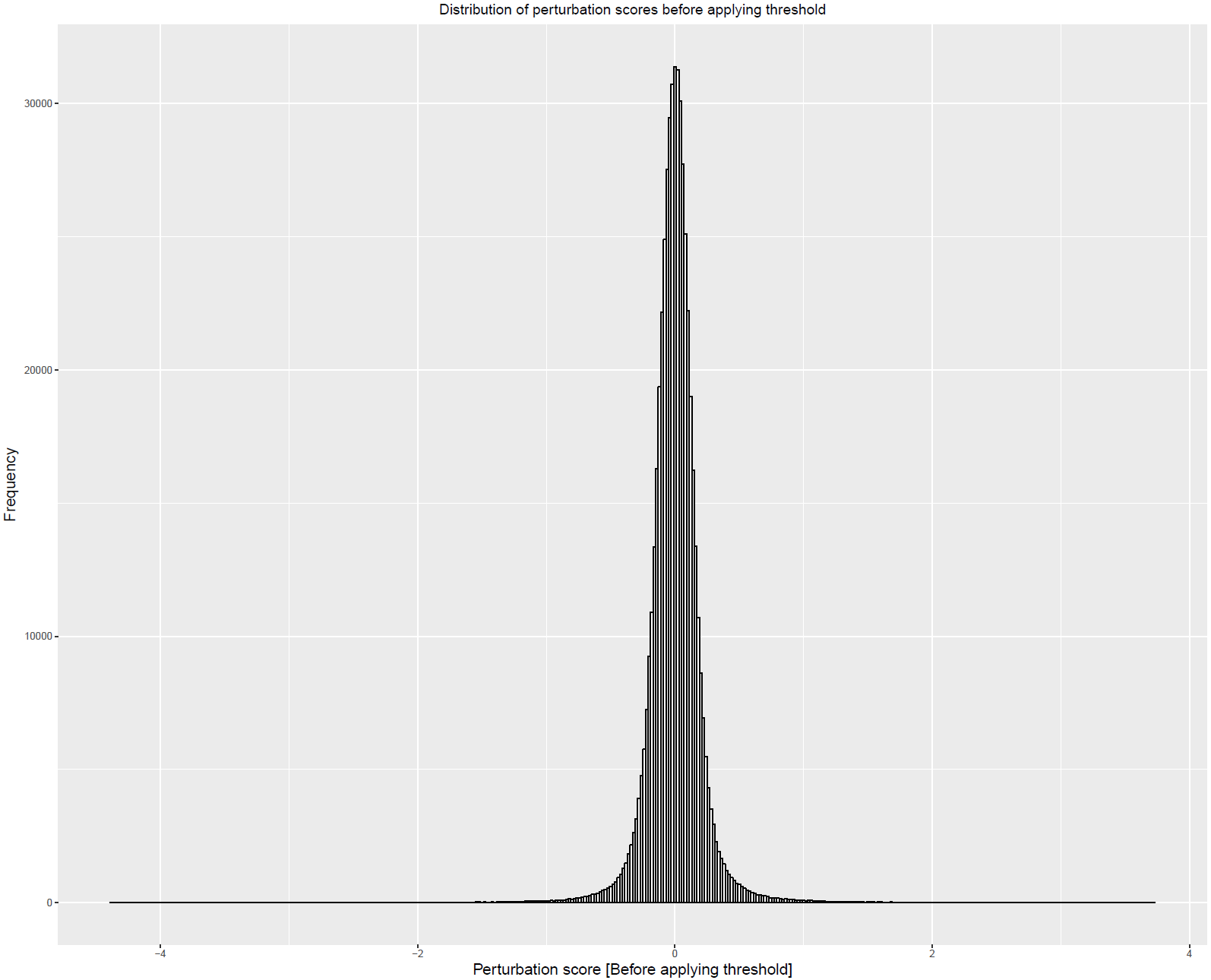


Supporting Figure 6. Distribution of the perturbation scores before applying cut-off threshold

Supporting Table 1. Network information for MSC senescence dataset

| Network | No. of node | No. of edge | Ratio of the used interaction (%) |
| --- | --- | --- | --- |
| Passage 2, 3 | 1613 | 2422 | 10.474 |
| Passage 4, 5 | 1184 | 1496 | 6.469 |
| Passage 6, 7, 8 | 201 | 175 | 0.757 |
| Passage 10, 11 | 1163 | 1512 | 6.539 |
| Common network | 37 | 23 | 0.099 |

# Threshold: ±0.7156

# Total number of used interactions for constructing network: 23,124.

Supporting Table 2. The result of statistical significance test to compare perturbation scores between two time points on MSC senescence dataset. We performed statistical test with a significance level of 0.05.

| Comparing time points | △P from our approach (mean) | △P from random sampling | | P-value |
| --- | --- | --- | --- | --- |
|  |  | (mean) | (standard deviation) |  |
| Passage 2,3 - Passage 4,5 | 1.186 | 0.799 | 0.146 | 1.163E-16 |
| Passage 4,5 - Passage 6,7,8 | 1.130 | 0.489 | 0.135 | 8.028E-50 |
| Passage 6,7,8 - Passage 10,11 | 1.037 | 0.477 | 0.136 | 1.430E-37 |
| Passage 2,3 - Passage 10,11 | 1.096 | 0.818 | 0.161 | 7.220E-08 |

Supporting Table 3. On the common network from MSC senescence dataset, the list of top 10 terms of functional enrichment test with KEGG pathway and Gene Ontology database. (P-value < 0.01)

| Category | Term | -Log_10_ (P-value) |
| --- | --- | --- |
| KEGG pathway | Cell cycle | 3.854 |
|  | Pathways in cancer | 3.796 |
|  | Colorectal cancer | 3.886 |
| Gene Ontology  (Biological Process) | cell cycle process | 10.718 |
|  | negative regulation of cellular process | 10.493 |
|  | mitotic cell cycle | 10.474 |
|  | cell cycle | 10.054 |
|  | negative regulation of biological process | 9.733 |
|  | cell cycle phase | 8.559 |
|  | regulation of cell cycle | 8.446 |
|  | regulation of mitotic cell cycle | 8.248 |
|  | gland development | 7.894 |
|  | spindle checkpoint | 7.582 |

Supporting Table 4. List of the number of selected genes for each time-point varying SD cut-off threshold

| Cut-off threshold | Time-point of replicative senescence dataset (GSE41714) | | | |
| --- | --- | --- | --- | --- |
|  | Early | Middle | Advanced | Very Advanced |
| μ ± 1SD | 6,739 | 3,418 | 2,015 | 4,469 |
| μ ± 2SD | 2,538 | 909 | 401 | 1,199 |
| μ ± 3SD | 1,167 | 378 | 154 | 516 |

Supporting Table 5. List of the attributes for the each time-point specific network and the common network varying SD cut-off threshold

| Cut-off threshold | Attributes | Time-point of replicative senescence dataset (GSE41714) | | | | Common network |
| --- | --- | --- | --- | --- | --- | --- |
|  |  | Early | Middle | Advanced | Very Advanced |  |
| μ ± 1SD | # Edge | 2,602 | 701 | 266 | 1,448 | 95 |
|  | # Node | 1,748 | 650 | 302 | 1,085 | 122 |
| μ ± 2SD | # Edge | 493 | 50 | 12 | 136 | 3 |
|  | # Node | 483 | 71 | 20 | 172 | 6 |
| μ ± 3SD | # Edge | 145 | 12 | 0 | 28 | 0 |
|  | # Node | 171 | 17 | 0 | 47 | 0 |

Supporting Table 6. The results of the multiple comparisons by ANOVA and TukeyHSD in R. P-values were obtained that present how the distribution of final perturbation scores differs between the two time-points in gene level.

| Comparisons | P-value |
| --- | --- |
| Early-Middle | 0.0410 |
| Early-Advanced | 0.0080 |
| Early-Very advanced | 0.0037 |
| Middle-Advanced | 0.9503 |
| Middle-Very advanced | 0.8726 |
| Advanced- Very advanced | 0.9961 |

**References**

1. Wagner, W. et al. Replicative senescence of mesenchymal stem cells: a continuous and organized process, PLoS One, 2008; 3: e2213.
